# Palliative care symptoms of people living with rapidly progressive prion diseases: a systematic review

**DOI:** 10.1186/s12904-026-02107-y

**Published:** 2026-04-27

**Authors:** Rachel T. Williams, Nicola White, Joshua Kearns, Kirsty McNiven, Ann Rochelle Marasigan, Elizabeth L. Sampson, Catherine J. Evans, Simon Mead, Nuriye Kupeli, the EMBED‐Care Programme, E L Sampson, C J Evans, K Sleeman, N Kupeli, K Moore, N Davies, C Ellis‐Smith, J Ward, A Gola, B Candy, Omar, J Warren, J Anderson, R Harding, R Stewart, S Mead

**Affiliations:** 1 MRC Prion Unit at UCL, Institute of Prion Disease, Courtauld Building, 33 Cleveland Street, London, W1W 7FF UK; 2 National Prion Clinic, University College London Hospitals NHS Foundation Trust, London, UK; 3 Marie Curie Palliative Care Research Department, Division of Psychiatry, UCL, London, W1T 7NF UK; 4 Centre for Psychiatry and Mental Health, Wolfson Institute of Population Health, Queen Mary University of London, Yvonne Carter Building, Turner Street, London, E1 2AB UK; 5 Academic Centre for Healthy Ageing (ACHA), Whipps Cross University Hospital, Barts Health NHS Trust, Whipps Cross Road, London, E11 1NR UK; 6 Florence Nightingale Faculty of Nursing, Midwifery & Palliative Care, Cicely Saunders Institute, King’s College London, Bessemer Road, London, SE5 9PJ UK; 7 School of Social Sciences, Education and Social Work, Queen’s University Belfast, Belfast, BT71NN UK

**Keywords:** Creutzfeldt-Jakob disease, Prion Disease, Palliative care

## Abstract

**Background:**

Creutzfeldt-Jakob disease (CJD) is a rapidly progressive, fatal dementia with an average prognosis of four to six months. Palliative care, as a holistic person-centred approach to supporting people and families affected by life-limiting illness, may be beneficial. However, despite the rapid disease trajectory, relatively little is known about the palliative care symptoms and concerns of this population. This systematic review identifies and reports the palliative care symptoms of people living with sporadic, iatrogenic, and acquired CJD.

**Method:**

Studies were included if they reported the symptoms of patients with a diagnosis of CJD (excluding inherited prion disease), using quantitative methodology. No date range was used to narrow the search, non-English language and non-peer reviewed papers were excluded. Searches for relevant studies published before December 2023 were conducted across five databases (including EMBASE, CINAHL, and PubMed) by eight independent reviewers. Methodological quality appraisal was undertaken using the Hawker Quality appraisal tool. Data on study characteristics and reported symptoms, were extracted. Identified symptoms associated with CJD were collated, and Artificial Intelligence assisted semantic clustering was performed to group symptoms into categories, the reported symptoms were tallied to identify those most commonly reported across the literature. A palliative care lens was then applied to classify symptoms as physical, psychological, social, or spiritual. The review protocol was registered with PROSPERO (CRD42020194294).

**Results:**

The search yielded 8,503 unique citations; following title, abstract, and full-text screening, 30 papers were included in the final analysis, describing 4,434 participants. Across the included literature, 109 unique symptoms were identified, 374 in total when accounting for duplicates. Symptoms were categorised into 11 subsets: motor impairments (17.6%, 66/374 proportion of total number of symptoms); coordination, balance and vestibular issues (17.6%, 66/374); behavioural, psychiatric and emotional symptoms (23.5%, 88/374); speech and language disorders (10.1%, 38/374); visual and sensory disturbances (7.7%, 29/374); cognitive disorders (6.9%, 26/374); sleep disorders (6.6%, 25/374); other neurological symptoms (4.2%, 16/374); swallowing and nutrition issues (2.9%, 11/374); autonomic and sphincter dysfunction (1.3%, 5/374); and social symptoms (1.0%, 4/374).

**Conclusion:**

Findings align with established knowledge regarding the diverse and complex symptom burden in CJD, highlighting the potential challenges of symptom management. Motor and coordination impairments were most frequently reported, alongside behavioural and psychiatric disturbance. Limited evidence was identified regarding social or spiritual symptoms, quality of life, or symptom progression, as many studies focused primarily on improving early diagnosis. Further primary research is required to better understand the palliative care needs of people living with CJD to ensure comprehensive end-of-life care.

**Supplementary Information:**

The online version contains supplementary material available at 10.1186/s12904-026-02107-y.

## Introduction

Prion diseases are a group of rare, fatal and transmissible neurodegenerative diseases with no curative treatment. They can occur sporadically, genetically or be acquired in the diet or through medical or surgical procedures [1]​​. Despite the shared disease mechanisms, prion diseases are highly variable with a wide range of symptoms, duration of illness and age of onset, making the clinical course unpredictable and difficult to manage [2, 3]​. Sporadic Creutzfeldt-Jakob disease (sCJD) is the most common form of prion disease, accounting for 85–90% of new cases. Its annual UK incidence is 1–2 per million with a lifetime risk of 1 in 5000 [4]. sCJD has a median survival of five months from symptom onset to death, however the prognosis is highly variable, with a range of weeks to up to ten years [3]. ​The mean age of onset is 67 with a range from childhood to old age. sCJD typically presents as a rapidly progressive dementia with additional neurological features such as cerebellar ataxia, myoclonus and other movement disorders [5]. The acquired prion diseases include iatrogenic CJD and variant CJD (vCJD), which resulted from dietary exposure to contaminated beef. These forms of CJD typically have onset at a younger age, longer duration of illness and are often marked by psychological difficulties such as transient delusions and auditory and visual hallucinations [6, 7]. There have been 178 probable or definite vCJD cases in the UK, incidence peaked in the early 2000s with no new reported cases in recent years [8]. Inherited prion diseases account for approximately 10–15% of the total incidence and are caused by a genetic mutation in the *PRNP* gene which encodes prion protein. There are over 50 identified pathogenic mutations in *PRNP*, some of which have a distinctly slowly progressive phenotype.

As sCJD and vCJD are life-limiting conditions, clinical care primarily centres on symptom management and proactive palliative planning. Early referral to palliative care services is thought to be an important aspect of providing care by enabling rapid access to end-of-life expertise across a variety of settings [9].The World Health Organisation (WHO) define palliative care as total care for patients and their families facing a life-limiting disease, encompassing the spiritual, physical, psychological, and social care of the patient and family [10]. Palliative care specialists play a key role in advance care planning, facilitating access to benefits, managing symptoms, and providing bereavement support. However, due to the rarity of CJD, many palliative care professionals encounter few patients with the condition and may therefore have limited knowledge of their specific needs [9, 11]. It is acknowledged in the literature that there are challenges to providing palliative care for patients with CJD, such as the rarity of disease, rapidity of progression, variability of symptoms and duration of illness [12, 13]. CJD patients are known to present with complex symptoms, requiring high levels of support and multidisciplinary input [9, 11, 14]. A scoping literature review to understand current palliative pharmacological management of CJD identified a lack of good quality evidence to support symptom management at end of life [15–18]. These publications highlight the complex symptoms of the patient population, and an absence of consensus on how symptoms at the end of life should be managed. A key principle to understanding palliative care needs is to take a holistic approach, championed by Dame Cicely Saunders and reflected in the WHO definition of palliative care, which emphasises the multidimensional nature of suffering, encompassing, physical, psychological, social, and spiritual pain, arguing that all need to be addressed to alleviate suffering [10].

### Aims

This systematic review will apply a palliative care lens to the prion disease literature to explore the following questions:

1. What are the palliative care symptoms (physical, psychological, social and spiritual) of people living with a prion disease?
2. Do these symptoms impact the quality-of-life of people with prion disease?

## Methods

This systematic review follows the Preferred Reporting Items for Systematic Reviews and Meta-Analyses (PRISMA; guidelines. The protocol for this review was registered on PROSPERO (CRD42020194294) [19].

### Search strategy

We searched five databases, Cumulative Index to Nursing and Allied Health Literature (CINAHL), Embase, OVID, EBSCO MEDLINE, and PsycINFO from inception to December 2023 for relevant papers. A combination of search terms, including medical subject heading (MeSH) terms, was used for prion disease and symptoms and features, including separate terms for physical, psychological, social and spiritual symptoms (see supplement 1 for the terms used). Backward and forward citation searching was also undertaken to identify any relevant studies that may not have been retrieved from the database searches.

### Eligibility criteria

Predetermined eligibility criteria relevant to the review aims were developed and used to screen titles and abstracts and full-text papers (see Table 1). In summary, studies reporting quantitative data to describe symptoms experienced by people living with prion disease, specifically rapidly progressive forms of CJD such as sporadic, iatrogenic, or variant, were included. Inherited forms of prion disease were excluded from this review as their clinical features are distinct and the duration of illness often spans over many years, with diverse care needs, depending on the specific *PRNP* mutation and phenotype.

**Table 1:** Inclusion and exclusion criteria used for screening relevant studies

Included studies	Excluded studies
Studies reporting data on people who have a formal confirmed or probable diagnosis of prion disease types: Sporadic, iatrogenic, variant or acquired	Participants with genetic or inherited prion disease, those with an unclear diagnosis or participants with a secondary cause of cognitive impairment such as post-stroke or traumatic brain injury that have caused prion symptoms
Studies reporting quantitative data (including mixed methods where quantitative data is reported) on physical, psychological, social or spiritual symptoms or palliative care symptoms/concerns of prion disease (i.e., cohort, observational, RCTs, diagnostic, case studies [where multiple cases are reported], retrospective case note review)	Publications reporting single cases, data synthesis, only qualitative data, editorial/opinion pieces, conference abstracts, theses
All settings included (i.e., home, care home, hospital)	Studies that do not report quantitative data (i.e., studies that only report qualitative data)
	Non-peer-reviewed studies or studies not published in English

Quantitative studies were prioritised based on a best‑evidence approach aligned with the review’s aims: to identify palliative care symptoms in prion disease, categorise them within a palliative care framework, and examine reported impacts on quality of life. These objectives required evidence that systematically measured symptom prevalence, frequency, or severity and, where available, their association with quality‑of‑life outcomes.

### Study selection

Titles and abstracts were reviewed for eligibility using the inclusion/exclusion criteria by multiple independent reviewers (RW, NK, ES, NW, JK, AM, KMN). To ensure consistent interpretation and application of the inclusion/exclusion criteria across the reviewers, all reviewers initially screened the same random sample of 100 titles and abstracts and met to discuss the results and check mutual understanding of the eligibility criteria. Abstracts were divided between the reviewers (RW, NK, ES, NW, JK, AM, KMN) and a second reviewer was assigned to ensure each abstract was screened independently twice. The inclusion and exclusion criteria were embedded in the screening form, and reviewers selected predefined outcome options. The same process was repeated for full text screening, the selected papers were divided between the reviewers (RW, NK, ES, NW, JK, AM, KMN) and a second marker was assigned, reviewers selected options from a predefined list to record their reasoning for exclusion where applicable. The results from both reviewers were compared for consensus. In cases of disagreement, a member of the group outside of the pair of reviewers screened the full text and consensus was reached through discussion. Authors of studies where additional information was required to inform decision making were contacted.

### Data extraction and analysis

The full text papers selected for inclusion were divided between the reviewers (RW, JK, AM, KMN) for data extraction. An excel spreadsheet was created to extract data on the design, study aim, methods including setting, measure/tool used to assess symptoms, population, and sample size, sample characteristics, symptom information, and quality of life data. Following data extraction the reviewers (RW, KMN, AM, JK) met to discuss the studies selected and to present the findings to the group.

The Hawker Quality appraisal tool [20] was used during data extraction of full text articles to assess the quality of the studies included. The Hawker quality appraisal tool is a systematic framework for evaluating the quality and validity of research studies, covering key aspects like study design, sample selection, data collection, analysis, and result interpretation, applicable to quantitative, qualitative, and mixed-methods studies [20]. Four independent reviewers (RW, JW, ARM, and KMN) assessed the methodological quality of the included studies and met to discuss the rating process to ensure consistency. Given the exploratory nature of the review, no studies were excluded on the basis of methodological quality. Each study was assigned an overall score representing poor (⩽18), fair (19 to 27) and good (⩾28) methodological quality.

### Symptom data and expert-assisted AI sematic clustering

We extracted symptom data from the included studies, each unique symptom reported was listed and the number of studies reporting the symptom was recorded. Due to the diverse range of studies included and the large number of symptoms reported, it was not possible to report on the prevalence of a symptom within each study, we instead reported the proportion of studies observing a particular symptom to get a sense of how frequently this symptom was identified. To manage the anticipated heterogeneity and volume of symptom descriptors reported in the literature, we used an expert-led semantic classification process supported by AI-assisted clustering. The extracted symptom terms were retained verbatim and analysed using ChatGPT (version 4o) as a decision-support tool to propose candidate groupings based on lexical similarity, synonymy, and contextual usage. These AI-suggested clusters were then reviewed, refined and, where necessary, re-assigned by prion disease clinicians (SM, RW), who applied predefined clinically meaningful categories and made the final classification decisions. The final symptom categories were subsequently mapped, through expert judgement, to the palliative care domains (physical, psychological, social and spiritual). The symptom data were then displayed in a sunburst chart to visualise the heterogeneity of the symptom data. All symptoms reported were displayed within their symptom category and the palliative care classification (physical, psychological, spiritual and social). The proportion of the sunburst chart occupied was calculated by tallying up the total number of studies reporting a particular symptom, the symptoms with larger sections were the most frequently reported in the studies reviewed.

## Results

**Fig. 1:**
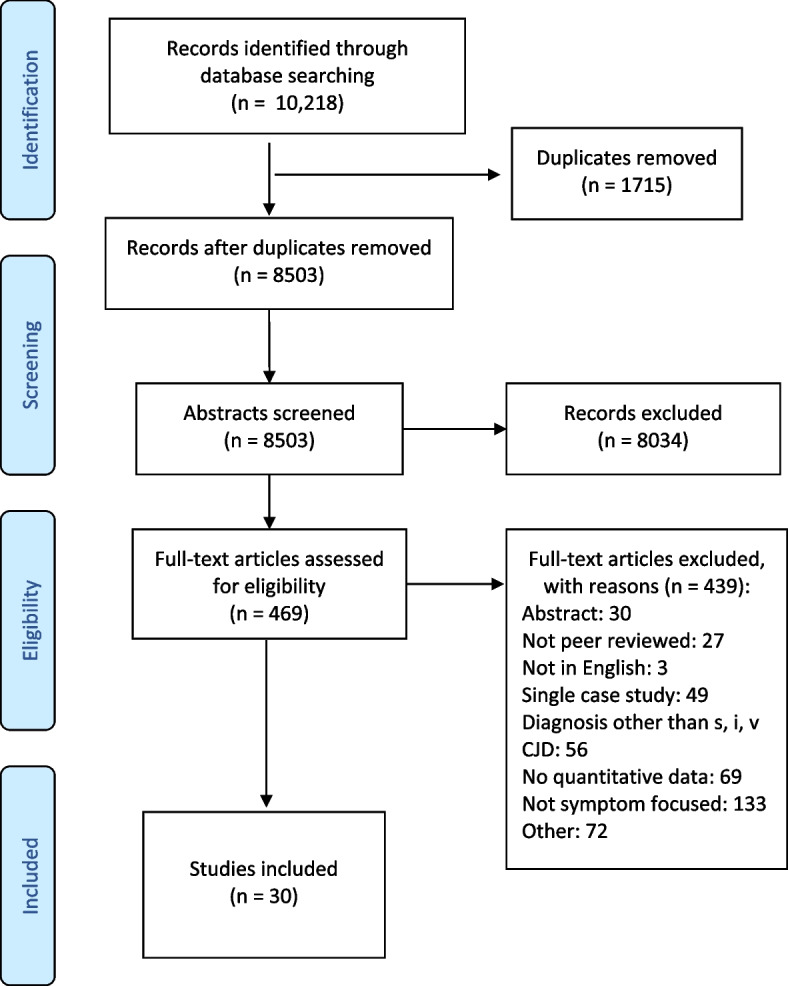
Prisma flowchart

The database searches identified 10,218 records, of which 1715 were then excluded as they were duplicates. A total of 8503 abstracts were screened, of which 8034 were excluded at this stage, 347 of these were additional duplicates. Full text screening of 469 papers resulted in the exclusion of 439 papers for the following reasons: Abstract: 30, Not peer reviewed: 27, Not in English: 3, Single case study: 49, Diagnosis other than sporadic, iatrogenic or variant CJD: 56, No quantitative data: 69, Not symptom focused: 133, Other: 72. Examples of reasons in the “Other” category were; review article, no full text available, book chapter, or duplicate. Following identification, screening and eligibility assessment, 30 studies were selected for inclusion. The included studies comprised cohort studies (18/30) [7, 21–38] retrospective case reviews (10/30) [39–48], and one retrospective case series [49] (Fig. 1).

### Sample

#### sCJD

The age of onset and disease duration of sCJD was found to be highly variable in most of the studies, except for those studies that had selected specific age groups as a primary focus (see Supplementary Table 2. for a summary of study characteristics). The average age reported ranged from 55—70 years [46, 50] with the majority of studies reporting the average age of onset to be in the 60s. [21, 28, 30–32, 39, 42, 43, 45, 48] The range reported for the averages was wide with the youngest age of onset reported as 19 years old and the oldest reported as 90 years old [39, 48].

The median duration of illness varied in studies reporting this data. The sCJD studies reported a range of median durations from 4.2 months [45]to 14.5 months [50]_,_ with the majority of the other studies reporting between 5 and 8.5 months [28, 30, 42–44, 47]. In studies also reporting the range of the duration of illness, wide variability was reported, with duration of illness reported as short as 2 months in some cases and as long as 41 months in other cases [28, 30, 45, 50].

#### vCJD

vCJD was reported as having a younger onset and a longer illness duration, with a median age of onset reported as between 26 to 29 years [7, 34, 37] with a range of 12 to 74 years, with a median duration of illness was 14 months [7, 33, 35–37].

### Focus of research

The studies included in this review were selected because they described the physical, psychological, spiritual or social symptoms of CJD. The majority described the physical symptoms of sCJD along with the psychological symptoms, however the aim of most of the studies was not to use the information gathered about symptoms to make recommendations regarding management but more to understand how these symptoms may help with diagnosis by highlighting the variability of presentation across differing ages, ethnic groups and genetic profiles.

The primary focus of most studies was centred on sporadic CJD, although some studies compared sCJD to other diseases. Edler et al. compared sCJD to other neurodegenerative diseases such as Alzheimer’s disease and Lewy body dementia to characterise the differing movement disorders between the conditions for diagnostic application along with the effect of codon 129 status in sCJD [30]. Dai et al. used polysomnography to compare sleep disturbance in sCJD to sleep disturbance in fatal familial insomnia (an inherited form of prion disease) [45].

As this systematic review did not stipulate a date range, some of the papers selected for inclusion were published at the time of the vCJD epidemic when the public health risk was not fully understood, this necessitated a focus on how to quickly diagnose the condition by describing the psychiatric and neurological features and how they were distinct from sCJD [7, 34–38, 48]. This research continued into the following years, Boesenberg et al. compared psychological and neurological symptoms of young and elderly patients with sCJD and patients with variant CJD [29], whilst Heath et al. specifically examined the diagnostic criteria of variant CJD to explore the possibility of a timelier diagnosis [33].

The aim of several studies was to characterise and analyse the clinical features of sCJD in order to report the supporting diagnostic and histopathological findings [31, 32, 39, 40, 42–44, 49]. Differing clinical presentations of subtypes of sCJD and associations with genetic factors, namely codon 129 genotype, were the focus of studies by Krasnianski et al. and Baiardi et al. [22, 23, 26, 28]. Appleby et al. and Langlands et al. studied the differing presentations of sCJD in racial and ethnic groups [24, 41]. Tam et al. examined the presentation and symptoms of those diagnosed with sCJD under the age of 50, and Karch et al. examined how the diagnostic profiles of late onset sCJD differed from younger patients with sCJD [21, 25]. Only one study specifically investigated the palliative care needs of patients with sCJD, utilising both cohort study findings and post-death carer interviews to aim to develop a neuropalliative pathway to better support patients with sCJD [50].

### Methodological quality appraisal

Table 2 presents the methodological quality of each of the included studies. All included studies were categorised as “good” with a score of 28 or over [20].

**Table 2:** Hawker quality appraisal table

Author, date	Abstract & Title	Introduction & Aims	Method & Data	Sampling	Data Analysis	Ethics & Bias	Findings/Results	Transferability/Generalisability	Implications & Usefulness	Study quality (Hawker tool)
Iwasaki Y, et al., 2012, Japan [39]	4	4	3	3	3	2	4	3	3	29/36
Karch A, et al., 2014, Germany [21]	4	4	4	3	4	3	4	3	4	33/36
Khan S, et al., 2021, Pakistan [40]	4	4	3	3	3	3	4	4	4	31/36
Krasnianski A, et al., 2006, Germany [22]	4	4	4	3	4	3	4	3	4	32/36
Krasnianski A, et al., 2006, Germany and UK [23]	4	4	4	3	4	3	4	4	4	33/36
Langlands G, et al., 2021, United Kingdom [24]	4	4	4	3	4	3	4	3	4	33/36
Rajalingam P, 2023, Australia [49]	4	4	4	3	4	3	4	3	4	32/36
Tam J, et al., 2022, United Kingdom [25]	4	4	4	4	4	3	4	4	4	35/36
Krasnianski A, et al., 2017, Germany [27]	4	4	4	4	4	3	4	4	4	35/36
Krasnianski A, et al., 2014, Germany [26]	4	4	4	4	4	3	4	4	4	35/36
Appleby B, et al., 2012, USA [41]	4	3	4	4	3	3	4	4	4	33/36
Baiardi, S., et al. 2017, Italy [28]	4	4	4	4	3	4	4	4	3	34/36
Boesenberg C, et al. 2005, Germany [29]	4	4	4	4	4	1	4	4	1	30/36
Brown P, et al., 1979, France [42]	3	4	3	4	4	1	4	4	3	30/36
Brown P, et al., 1986, France [43]	3	3	4	4	4	1	4	4	3	30/36
Shuai C., et al., 2020, China [44]	3	4	4	4	4	4	4	4	3	34/36
Dai Y., et al., 2021, China [45]	4	4	4	3	4	4	4	4	4	35/36
Drobny M., et al., 1991, Czecho-Slovakia [46]	3	3	3	4	4	1	4	4	3	29/36
Edler J., et al., 2009, Germany [30]	4	4	4	4	4	4	4	4	4	36/36
Feng S, et al., 2021, China [31]	4	4	4	4	4	4	4	4	4	36/36
Gao C, et al., 2011, China [32]	4	4	4	4	4	4	4	4	4	36/36
Gurram S, et al., 2023, India [47]	4	4	4	3	4	4	4	4	3	34/36
Harrison KL, et al., 2022, USA [50]	3	4	4	4	4	2	4	4	4	33/36
Heath CA, et al., 2010, UK [33]	4	4	4	4	4	4	4	3	3	34/36
Spencer, M, et al., 2002 UK [34]	4	3	4	4	4	4	4	3	4	34/36
Wall, C et al., 2005 UK [48]	4	3	4	4	4	4	3	3	4	33/36
Will, R et al., 1999 UK [35]	3	4	4	4	3	4	3	3	4	32/36
Will, R et al., 2000 UK [36]	4	4	4	4	4	4	4	3	4	35/36
Zeidler, M et al., 1997, UK [7]	4	3	4	4	4	4	4	3	4	34/36
Zeidler M et al., 1997, UK [37]	4	3	4	4	4	4	4	3	4	34/36

Key: Overall scoring represents Good: ≥ 28; Fair: 19–27; Poor: ≤ 18

### Palliative care symptoms

**Fig. 2:**
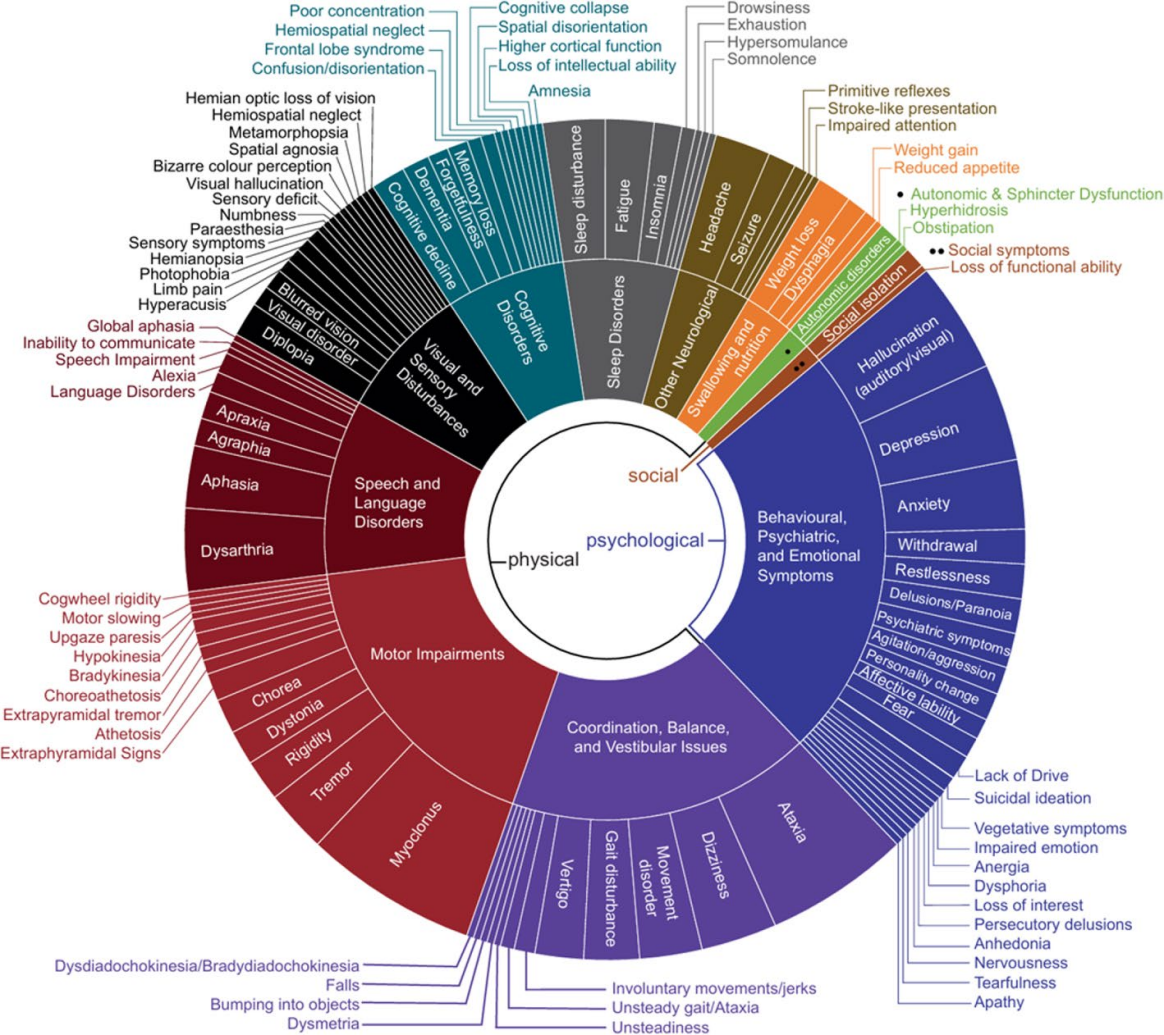
Symptom reporting and palliative care domains (physical, psychological, spiritual, and social), symptom category and individual symptom

The palliative care categories of physical, psychological, social, or spiritual symptoms are displayed in the centre of Fig. 2. The inner layer lists the symptom categories, and the outer layer lists the individual symptom within each category. The data used to create the Fig. 2 are included in the supplementary files, see Supplementary Table, 3: Symptoms list and tally.

### Symptoms chart and the palliative care lens

The classification of symptoms into discrete categories revealed distinct patterns of presentation, shedding light on the diverse phenotypic expressions of CJD. Semantic clustering resulted in the following 11 categories: Motor impairments (17.6%, 66/374 total number of symptom reports); coordination, balance and vestibular issues (17.6%, 66/374); Behavioural, psychiatric and emotional symptoms (23.5%, 88/374); Speech and language disorders (10.1%, 38/374); Visual and sensory disturbances (7.7%, 29/374); Cognitive disorders (6.9%, 26/374); sleep disorders (6.6%, 25/374); Other Neurological (4.2%, 16/374); Swallowing and nutrition (2.9%, 11/374); Autonomic and sphincter dysfunction (1.3%, 5/374) and Social symptoms (1.0%, 4/374). While physical symptoms dominated the literature (75.5%), the psychological and behavioural manifestations were also substantial (23.5%), highlighting the multidimensional burden of the disease.

#### Physical symptoms

There were 109 distinct symptoms observed across the literature. Because multiple studies described the same symptom, the total number of symptoms reported across all studies combined was 374. Motor impairments were among the most consistently reported symptoms, with myoclonus, a hallmark feature of CJD, emerging as the most prevalent, at 37.9% (25/66 proportion within symptom category). Myoclonus was often described as sudden, involuntary jerks that could occur spontaneously or be triggered by stimuli, reflecting the hyperexcitability of the nervous system in prion diseases. Ataxia, another frequently observed symptom within the coordination, balance, and vestibular issues category, contributed to 31.8% (21/66 proportion within symptom category) of the symptoms reported in this domain.

In addition to gross motor dysfunction, visual and sensory disturbances were also noted, with diplopia, visual misperceptions, and complex visual hallucinations featuring in some reports. These symptoms likely reflect occipital lobe involvement in CJD, particularly in cases with Heidenhain variants, where visual symptoms precede cognitive decline. Some studies also detailed paraesthesia and other altered sensory experiences (20.6%, 6/29), which, though less frequently reported, add to the complex neurological picture of CJD.

Speech and language disorders were another important subset of physical symptoms, ranging from dysarthria (slurred speech) 30.0% (12/38) to aphasia (language impairment) (22.5%, 9/38). In some patients, speech production declined in tandem with cognitive deterioration, while in others, isolated language difficulties, such as word-finding pauses or reduced verbal output, were among the initial symptoms.

#### Psychological, and social symptoms: hallucinations, emotional distress, and disinhibition

Psychiatric symptoms were common and often preceded neurological decline, reinforcing the diagnostic challenge of early-stage CJD. Hallucinations (17.8%, 16/88), depression (15.6%, 14/88), and anxiety (11.3%, 10/88) were the most frequently reported psychological symptoms, with hallucinations ranging from simple visual distortions to complex, distressing visions of people or objects. These symptoms were often accompanied by delusions, particularly paranoid or persecutory themes (6.7%, 6/88), which may reflect a combination of organic brain dysfunction and the patient’s awareness of their cognitive decline.

Affective symptoms, including apathy (1.1%, 1/88), withdrawal (5.6%, 5/88), lack of drive (2.2%, 2/88) and impaired emotion (1.1%, 1/90), were also reported.

#### Social symptoms

Social symptoms were notably underreported in the quantitative literature (1.1%, 4/374s), but when described, they highlighted profound isolation and loss of interpersonal engagement. Functional decline, often captured indirectly through descriptions of dependence in activities of daily living (ADLs), was a unifying feature across cases. Some studies noted a rapid transition from mild forgetfulness to complete loss of self-care ability within a few months, while others described more insidious declines in social interaction, withdrawal from conversations, and loss of insight.

#### Spiritual symptoms

The spiritual and existential impact of CJD is absent in most quantitative studies, highlighting an area requiring further exploration. Patients facing such an aggressive and incurable illness likely experience profound existential distress, but this aspect remains largely unexamined in the quantitative literature reviewed.

### Quality of life

None of the quantitative studies included in this review assessed quality of life in individuals diagnosed with CJD.

## Discussion

This review sought to explore the palliative care symptoms of people living with CJD by systematically assessing reported symptoms through the palliative care lens. The findings highlight the complexity and multidimensional nature of the disease but also reveal a gap in quantitative research regarding quality of life, social experiences, and spiritual well-being. Whilst physical symptoms, particularly myoclonus, ataxia, dysarthria, visual disturbances, and sleep dysfunction, were well documented, the review found limited reports of social and spiritual distress. Psychological and behavioural symptoms, including hallucinations, depression, anxiety, and disinhibition, were also widely observed, though they were often described in relation to diagnosis rather than in the context of patient care. This may be in part due to the focus on quantitative literature. It is possible that the inclusion of qualitative literature, which may be more commonly utilised to explore social and spiritual distress along with quality of life, may have yielded different results.

The application of the palliative care lens, which conceptualises suffering as a combination of physical, psychological, social, and spiritual pain, revealed that the most commonly reported aspects of suffering in CJD patients are physical and psychological symptoms. The model was useful in organizing and interpreting the physical and psychological dimensions of suffering, and for highlighting the absence of quantitative data on the social and spiritual aspects of the disease. Although it is evident to practitioners that patients with CJD experience profound existential distress, loss of identity, and social isolation, particularly in the early-middle stages of disease, these aspects remain somewhat unexplored in quantitative studies. The dominance of diagnostic-focused quantitative research has left gaps in understanding the lived experience of CJD and the true burden of suffering for both patients and their families.

When compared to previous qualitative research, particularly the work of Liz Ford et al. [18], the findings of this review highlight key discrepancies in how symptoms are understood. Ford and colleagues identified mobility and coordination difficulties, mood and behavioural symptoms, personal care, continence, eating and swallowing, communication, and cognitive impairment as the most distressing symptoms from a caregiver perspective. While this review confirms that these symptoms are frequently reported in the quantitative literature, issues such as continence, swallowing difficulties, and long-term functional decline were less frequently documented. This discrepancy likely reflects the different aims of the research; many studies included in this review were designed to define diagnostic subtypes rather than to examine how symptoms evolve and affect daily functioning. Few studies considered the impact of disease progression on patient autonomy, further illustrating the disconnect between clinical symptom reporting and real-world care needs.

The work of Harrison et al. [50] assessed the carer burden with a series of carer interviews after the death of their loved one but did not assess the patient quality of life. Uflacker et al. [51] have previously compared the carer burden of sCJD with other neurodegenerative diseases, finding that sCJD has a high carer burden and distress, possibly in relation to psychiatric symptoms. The high carer burden and distress may be a proxy for the patient experience but the lack of evidence regarding patient quality of life emphasises the importance of further research to understand quality of life in patients with CJD. Given the aggressive disease course and rapid loss of independence, it is likely that quality of life deteriorates profoundly, but without formal assessments, clinicians are left with little guidance on how to mitigate suffering or conduct health economic modelling. Understanding how quality of life changes over time in CJD could inform targeted palliative care interventions to address unmet needs.

## Strengths and limitations

### Limitations

The exclusion of qualitative studies, while important for methodological consistency, may have contributed to the absence of insights into social and spiritual symptoms, which are often better captured through caregiver narratives and patient interviews. However, the exclusion of qualitative data aligned with the study aims to capture the best evidence with measurable outcomes. Quality of life search terms were not used because our primary aim was to understand the palliative care symptoms and a secondary aim was to explore whether quality of life was described or assessed within the included papers. Therefore, there may be papers that assessed quality of life, but these would have been beyond the primary scope of this review. The AI-assisted approach used for semantic clustering of symptoms also required expert review, as some terms were erroneously omitted or grouped incorrectly, highlighting the need for human oversight in AI-driven analysis.

The heterogeneity of the symptom data complicated analysis and synthesis. We generated a long list of symptoms, but this was difficult to interpret due to overlap in terminology and differing aims and characteristics of the studies identified. We acknowledge that reporting the proportion of studies identifying a specific symptom has limitations and that if the study was focused on a particular symptom such as sleep, the author may not have reported other symptoms that were present.

Finally, the Hawker quality appraisal tool used in this review is a generic instrument and was not designed for any single study type. However, it enabled systematic evaluation and comparison of methodological quality across heterogeneous study designs. The majority of included studies were rated as good quality, although specific methodological domains within some studies were assessed as poor or very poor. Given the exploratory nature of this review and the methodological heterogeneity of the included literature, the findings should not be interpreted as constituting a definitive evidence base. Rather, the synthesis reflects the current state of an emerging field, and conclusions should be considered in light of the variability in study quality described above.

### Strengths

Comprehensive search terms were used to ensure relevant literature was screened for eligibility in the review. Independent reviewers screened and reviewed the studies, interrater testing was undertaken to check consensus across the group. The AI-assisted clustering was supported by expert discussion and consensus. The focus on quantitative research resulted in a clear view of the diverse number of symptoms experienced within the CJD disease course, highlighting the heterogeneous nature of CJD and the inherent difficulty planning care for this population.

### Implications for research, clinical practice, and policy

This review concludes with the need for dedicated palliative care research in CJD, moving beyond symptom classification to focus on patient-centred outcomes. Future research should incorporate standardized quality of life assessments to better understand how symptoms impact patients and caregivers over time. More attention should be given to social and spiritual well-being, as these aspects remain poorly studied despite their likely importance in shaping the overall experience of illness. The inclusion of qualitative and mixed-methods studies could provide valuable insights into patient and carer perspectives, offering a more holistic understanding of suffering in CJD. There is also a need for evidence-based neuropalliative pathways, integrating symptom management, psychosocial support, and anticipatory guidance to ensure that patients receive the best possible care throughout their disease trajectory [50].

By shifting the research focus from early diagnosis to holistic care, future studies can help address the profound and multidimensional suffering experienced by patients with CJD and their families. The findings of this review indicate that while much progress has been made in understanding the pathology of CJD and its associated symptoms and signs, within the quantitative literature little is known about the patient experience of living with CJD. Strengthening palliative care research in this area will be essential to improving the quality of care and ultimately ensuring that patients with CJD receive the comprehensive and compassionate support they need.

## Conclusion

The findings of this review highlight the large number of physical and psychological symptoms associated with rapidly progressive forms of CJD, emphasising the complexity of the disease and the subsequent need for palliative symptom management expertise. Whilst many physical and psychological symptoms were recorded, information on the social or spiritual symptoms of the disease was sparse. This may in part be due to the exclusion of qualitative studies from this review. In the context of palliative care, which uses a holistic approach, the findings from this review of quantitative literature give an incomplete view of the care needs of an individual with CJD, lacking understanding of the patient experience, quality of life and the challenges faced as the disease progresses. More quantitative research is needed to develop a nuanced understanding of the palliative care needs in this population, the findings of which could be used to develop guidelines for palliative care specialists to better support those affected by the disease and mitigate some of the associated distress.

## Supplementary Information

Supplementary Material 1: Supplementary Table 1: Search terms for prion disease and symptoms. Supplementary Table 2: Summary of study characteristics. Supplementary Table 3: Symptoms list and tally.

## Data Availability

All data generated or analysed during this study are included in this published article [and its supplementary information files].

## Abbreviations

AD: Alzheimer’s disease
ADLs: Activities of daily living
AI: Artificial intelligence
BSE: Bovine spongiform encephalopathy
CJD: Creutzfeldt-Jakob disease
CSF: Cerebrospinal fluid
CT: Computed tomography
DLB: Dementia with Lewy bodies
EEG: Electroencephalography
FFI: Fatal familial insomnia
iCJD: Iatrogenic Creutzfeldt-Jakob disease
IPD: Inherited prion disease
MeSH: Medical Subject Headings
MRI: Magnetic resonance imaging
NSE: Neuron-specific enolase
PRNP: Prion protein gene
PRISMA: Preferred Reporting Items for Systematic Reviews and Meta-Analyses
PROSPERO: International Prospective Register of Systematic Reviews
PSG: Polysomnography
RCTs: Randomised controlled trials
RT-QuIC: Real-time quaking-induced conversion
sCJD: Sporadic Creutzfeldt-Jakob disease
TSE: Transmissible spongiform encephalopathy
vCJD: Variant Creutzfeldt-Jakob disease
WHO: World Health Organization
