# Supplementary material for: Palliative care symptoms of people living with rapidly progressive prion diseases: a systematic review
Source: BMC Palliat Care. 2026 Apr 27;25:169. doi: 10.1186/s12904-026-02107-y (PMC13255439; doi:10.1186/s12904-026-02107-y)
Supplement: Supplementary file 1 — Supplementary Material 1: Supplementary Table 1: Search terms for prion disease and symptoms. Supplementary Table 2: Summary of study characteristics. Supplementary Table 3: Symptoms list and tally. [file 12904_2026_2107_MOESM1_ESM.docx]

## Supplementary files

**Supplementary Table 1:** Search terms for prion disease and symptoms

| **#** | **Term** | **Mesh/related terms** |
| --- | --- | --- |
| 1 | Prion disease | prion disease OR prion disorder OR human prion disease OR inherited Prion disease OR genetic Prion disease OR creutzfeldt-jakob disease OR CJD OR creutzfeldt-jakob syndrome OR sporadic creutzfeldt-jakob disease OR sCJD OR sporadic prion disease OR variant creutzfeldt-jakob disease OR vCJD OR iatrogenic creutzfeldt-jakob disease OR iCJD OR prion dementia OR Kuru OR transmissible spongiform encephalopathy OR TSE OR bovine spongiform encephalopathy OR BSE OR brain spongiosis |
| 2 | Symptoms/needs/features | Symptom Assessment/ OR symptoms OR “affective symptom*” or Affective Symptoms/ OR “behavioural symptom*” OR “cognitive symptom” or Neurobehavioral Manifestations/ |
| 3 | Physical | “activities N2 physical” OR “mobility limitation” or Mobility Limitation/ OR physical OR Pain/ OR dyspn*ea or Dyspnea/ OR drowsiness OR ulceration OR skin or Skin/ OR “sensory loss” OR Paresthesia/ or par*sthesia OR aspiration OR ataxia or Ataxia/ OR dyspraxia or Apraxias/ OR “cortical  blindness” or Blindness, Cortical/ OR photosensitivity OR vision or Vision, Ocular/ OR “activities of daily living” OR ADLs OR dysphasia or Aphasia/ OR Dysarthria/ or dysarthria OR perseveration OR “mobility symptoms” OR myoclonus or Myoclonus/ OR aggression or Aggression/ OR distress OR agitation OR restlessness or Psychomotor Agitation/ OR repetition OR changes N2 eating OR changes N2 drinking OR apraxia or Apraxias/ OR hypersalivation or Sialorrhea/ OR “circadian rhythm disruption” OR “fatal familial insomnia” or Insomnia, Fatal Familial/ OR “urinary frequency” OR incontinence or Urinary Incontinence/ or Fecal Incontinence/ OR seizure or Seizures/ OR Nausea/ or nausea OR Vomiting/ or vomiting |
| 4 | Psychological | psychological or Psychological Distress/ OR Psychological Distress OR apathy or Apathy/ OR disinhibition OR behaviour OR Memory/ or memory OR communication or Communication/ OR “attention deficit” OR “executive dysfunction” OR “visuospatial awareness” OR Hallucinations/ or hallucinations OR delusions or Delusions/ OR “mood disturbances” OR Mood Disorders/ or Anxiety/ or Depression/ OR “mood disturbances” OR psychosis or Psychotic Disorders/ OR Cognitive Dysfunction/ or Executive Function/ OR Perceptual Disorders/ or “visuospatial awareness” OR “attention deficit” or Attention/ |
| 5 | Social | “social adjustment” or Social Adjustment/ OR Social Alienation/ OR Social Behavior Disorders/ or “social behavior* disorder*” OR Social Behavior Disorders/ OR Social Behavior/ OR Social Cognition/ OR social OR “cultural expectation” OR “social interaction*” OR “social connection” OR “social value” |
| 6 | Spiritual | spiritual or Spiritual Therapies/ or Spirituality/ OR religion or Religion/ OR ((relig* or spirit* or pray* or prey* or pastoral* or belief* or believe* or heal or healing* or faith* or multifaith* or multi faith* yog*) N5 (interven* or prog* or ritual* or car* or service* or plan* or resourc* or attitude* or need* or aspect* or nurs* or support* or therap* or help* or assist* or treat* or ceremon*)) OR spiritual* or religio* OR deity or divinity or divine OR church* or cleric or clergy* or priest* or preacher* or vicar* or minister* N10 religi* or minister N10 church OR shamanism or mystic* or transcend*or esoteric OR existential or salutogenesis OR Buddhis* or Christian* or catholic* or jew* or muslim* or muslem* or moslem* or "eastern orthodoxy" or "Jehovah* witness*" or protestant* or Hindu* or Islam* or Judaism or Tao* or Sikh* or Rastafari* or theology OR confucianism or mystic* or "eastern philosophy" OR God or "supreme being" or "higher being" |

**Supplementary table 2:Summary of study characteristics**

| **Author, date** | **Study design** | **Study aim** | **Sample characteristics** | **Method of diagnosis** |
| --- | --- | --- | --- | --- |
| Appleby B et al, 2012, USA (41) | Retrospective clinical review of cases | This study aims to examine differences in demographic, clinical, diagnostic, genetic, and neuropathological characteristics among CJD patients in different racial/ethnic groups. | 116 patients with diagnosis of probable or definite sCJD | Clinical evaluation, MRI, CSF, genetic analysis |
| Brown P, et al, 1979, France (42) | Retrospective review of clinical cases | This study aims to analysed the clinical characteristics of all histopathological verified cases found in France during the decide 1967-1977. | 62 male: 62 female. Mean age at onset 60 +/- 9 years, majority of patients between 55-75year (77%), peak frequency occured60-64 year age group. Mean duration of illness 8.5 months. All cases diagnosed as CJD: autopsy = 94; biopsy only = 30. | Clinical evaluation, histopathology |
| Brown P, et al, 1986, France (43) | Retrospective review of clinical cases | This study presents clinical analysis of a consecutive series of 230 neuropathologically verified CJD cases in France, from 1968 to 1982. | 230 patients; male: female ration 0.83; age at onset 19-83 years (mean 61.5 years), mean duration of illness was 7.6 months. | Clinical evaluation, histopathology |
| Dai Y., et al, 2021, China (45) | Retrospective review of clinical cases | To summarize the clinical characteristics of patients with sporadic Creutzfeldt-Jakob disease (sCJD), analyse its sleep disorder characteristics using polysomnography (PSG), and compare sleep disturbances with those of fatal familial insomnia (FFI). | 5 male, 4 female, average age at onset 61 years (53-70 years). Average disease course 4.2 months (2-9 months). | Diagnostic criteria for sCJD (MRI-CJD Consortium Diagnostic Criteria, 2009) |
| Drobny M., et al, 1991, Czecho-Slovakia (46) | Retrospective review of clinical cases | This study aims to Evaluate of the early, late and terminal clinical features of CJD, based on an analysis of the clinical histories and clinical pictures of 12 cases occurring in 3 groups. | 12 patients; 5 males, 7 females. Average age 54.5 years (35-70 years). 10 definite sCJD, 2 probable sCJD. | Clinical histories and clinical pictures. Clinical picture division based on Jansen and Monrad-Krohn. |
| Gurram S, et al, 2023, (47) | Retrospective case review of clinical cases | This study aims to characterize the clinical, radiological features and the outcome of patients with CJD with movement disorders as the forthcoming manifestation. | "25 patients (13 males) of sCJD with median age at presentation of 58 years and median duration of illness of 5 months were included in the study. 1 patient was classified as definite sCJD, 20 as probable and 2 as possible CJD. " | Magnetic resonance imaging of brain, Electroencephalography, Cerebrospinal fluid 14-3-3 assay. |
| Iwasaki Y, et al, 2012, Japan (39) | Retrospective case review of clinical cases | To investigate the accuracy of clinical diagnosis of Creutzfeldt–Jakob disease (CJD) in autopsy-confirmed cases | Number of Cases: 56 autopsy-confirmed CJD cases (3 of which not clinically diagnosed); Sex: 32 males (57.1%), 24 females (42.9%); Age at Onset: Average 66.4 ± 11.0 years (range 27–89 years) | Diagnostic criteria for CJD used to select and classify participants |
| Khan et al., 2021, Pakistan (40) | Retrospective review of clinical cases | To review and document cases of sporadic CJD (sCJD) in Pakistan and evaluate diagnostic limitations | 11 patients (48-76 years; mean 62 ± 9 years; 54% male) | European MRI-CJD consortium criteria; Brain MRI; EEG; CSF testing; Brain biopsy (if available) |
| Shuai C., et al, 2020, China (44) | Retrospective review of clinical cases | The study presents clinical analysis of 26 cases of sCJD | sCJD patient n=26; male/female=14/12; age at onset 57.7+/- 9.6 (27-60). Survival time (months) 7.7 (2-26) | sCJD criteria by China Centre for Disease Control and Prevention |
| Wall, C et al, 2005 UK (48) | Retrospective review of clinical cases | This study explores the reported frequency, timing, and treatment of psychiatric symptoms during the disease course of sporadic CJD. | 126 met criteria for either definite (N=49) or probable (N=77) CJD. No vCJD, 9 Inherited Prion Disease. The mean age 62 years, range of 30.0 to 90.7. onset to diagnosis 4 months, onset to death 6 months | Computed tomography (CT) and magnetic resonance imaging (MRI), EEGs, Neuron Specific Enolase (NSE) and 14–3–3 assays in the cerebral spinal fluid (CSF) were recorded when available. |
| Baiardia, S., et al 2017, Italy (28) | Cohort study | To identify clinical features and biomarkers that facilitate the early diagnosis of sporadic CJD VV2 | n=120 sCJD VV; n=93 sCJDVV2, n=27 probable sCJD at post-mortem. 64 female, 56 male, mean age at clinical onset 64.1 (39-82 years), mean disease duration 6.4 months (2-20 months) | Post-mortem examination and molecular consensus criteria (93); Diagnostic criteria for 'probable' sCJD (27) |
| Boesenberg C, et al 2005, Germany (29) | Cohort study | To describe the psychiatric and neurological features of these young patients with emphasis on the different codon 129 genotypes and PrP types, and to compare them with elder patients with sCJD and patients with variant CJD. | 52 definite and probable sCJD. Patients had to be 50 years or younger when the first symptoms occurred. Iatrogenic and genetic cases were excluded. For comparison, data on patients who were older than 50 years at disease onset included. | Clinical features, MRI, EEG, CSF RT-QuIC, 14-3-3, PRNP sequencing and neuropathological findings were examined. |
| Edler J., et al, 2009, Germany (30) | Cohort study | To determine the characteristics and frequencies of movement disturbances in CJD, AD, and DLB and to discover whether these diseases are distinguishable based on these observations. In addition, the influence of the codon 129-polymorphism of the prion protein gene on movement disturbances in CJD patients is investigated. | 143 patients - CJD n=100, AD n=29, DLB n=7, other n=7. CJD patients - 46M/54F, mean age at onset 67 (24-88), average disease duration in months 7 (2-23), MM 64, MV 17, VV 12 No record 7. Data on control comparisons available. | WHO criteria for sCJD. Clinical evaluation, histopathology |
| Feng S, et al, 2021, China (31) | Cohort study | To characterize the epidemiological and clinical characteristics of sporadic Creutzfeldt–Jakob disease (sCJD) in eastern China | 67 sCJD; 62 prob CJD, 5 poss CJD. 28 male, 39 female, ratio 1:1.39. Mean age 64.42 ± 9.00 years (range: 29–88 years) | "Brain MRI, 14-3-3 protein in CSF, and EEG |
| Gao C, et al, 2011 China (32) | Cohort study | The aim is to comparably describe the epidemical, clinical and laboratory features of Chinese CJD patients based on this surveillance | " 261 sCJD patients, 146 were males and 115 were females, with the gender ratio of 1.27:1; onset age of prob sCJD ranged from 21-82y, median age of 61. Poss sCJD from 18-81y, median age of 60. " | PRNP sequencing analysis, Western immunoblot (14-3-3 protein), brain tissue autopsy biopsy applied into neuropathologic assays and/or PrPsc detection w/ immunohistochemistry and/or western blot |
| Heath CA, et al, 2010 UK (33) | Cohort study | The aim is to explore the diagnostic process of vCJD in detail and to determine whether earlier diagnosis is possible " | 106 cases (definite cases) , 44 clinically probable. Of 150 cases, 84 were male and 66 were female. Median age at onset was 26 years (mean 28, range 12e74 years) , median age at death 28 years (mean 30.1; range 15e75 years). Median duration of illness was 14 months (mean 15.7; range 6.5e40 months). One case remains alive, 96 months after onset. | Cortical biopsy, tonsil biopsy, MRI |
| Krasnianski A, et al, 2006, Germany (22) | Cohort study | To describe clinical features and diagnostic tests of the MM2 cortical subtype in sporadic Creutzfeldt-Jakob disease | 12 patients with genetically and neuropathologically verified MM2 cortical sCJD | Clinical symptoms, MRI, EEG, CSF markers (S100B, 14-3-3 protein) |
| Karch A, et al., 2014, Germany (21) | Cohort study | To evaluate clinical, CSF, EEG, and MRI features of sCJD in patients older than 75 years compared to those younger than 75 years | 73 patients older than 75 years, 73 patients younger than 75 years | Clinical symptoms, CSF markers, MRI, EEG |
| Krasnianski A et al., 2006, Germany and UK (23) | Cohort study | To improve the diagnosis of patients with the MV2 subtype of sporadic Creutzfeldt-Jakob disease | 26 patients with MV2 subtype of sCJD | Clinical symptoms, MRI, EEG, CSF markers (tau-protein, 14-3-3 protein) |
| Krasnianski A et al., 2017, Germany (27) | Cohort study | To perform a detailed evaluation of neuropsychological deficits in a large group of definite sCJD patients with known molecular subtype | 248 patients with definite sCJD, known M129V polymorphism, and PrPSc type. 140 female, 108 male | Clinical symptoms, MRI, EEG, CSF analysis, genetic testing, neuropathology |
| Krasnianski A et al., 2014, Germany (26) | Cohort study | To describe the first symptom/sign and first diagnosis in patients with sporadic CJD in Germany with respect to M129V polymorphism and prion protein type | 492 patients with probable and definite sCJD and known M129V polymorphism | Clinical symptoms, CSF analysis, EEG, MRI, genetic testing, neuropathology |
| Langlands G et al., 2021, United Kingdom (24) | Cohort study | To ascertain the proportion of sCJD cases with non-White ethnicity in the UK and compare clinical and investigation findings between non-White and White cases | 1697 CJD Cases - 1642 | Clinical symptoms, CSF 14-3-3, RT-QuIC, EEG, MRI, codon 129 genotyping |
| Spencer, M, et al, 2002 UK (34) | Cohort Study | To describe the early psychiatric and neurological features of variant Creutzfeldt-Jakob disease. | 100 patients, median age at onset was 26 (range 12-74) years. median duration of illness was 13.0 (6-39) months. | Met diagnostic criteria for diagnosis of variant CJD, NCJDRSU surveillance unit |
| Tam J et al., 2022, United Kingdom (25) | Cohort study | To characterize clinical, investigation, and neuropathological features in young individuals with sCJD | 46 young (≤50 years) out of 1178 sCJD cases | Clinical symptoms, MRI, EEG, CSF RT-QuIC, 14-3-3, PRNP sequencing, neuropathology |
| Will, R et al, 1999 UK (35) | Cohort study | This study aims to describe the early psychiatric symptoms of variant CJD and how they could lead to diagnosis of common psychiatric diseases. | 33 confirmed cases of vCJD, mean age at death of 30 (18-53) | MRI, EEG, psychiatric and neurological assessment. |
| Will, R et al, 2000 UK (36) | Cohort study | The aim is to develop a diagnostic criteria for the diagnosis of nvCJD are proposed, and data on the sensitivity and specificity of these criteria are presented. | 33 cases neuropathologically confirmed and 2 classified as probable nvCJD. Fifteen cases were male and 20 female. The median illness duration was 14 months (range, 8 –38 months) and the median age at death was 29 years (range, 18 –53 years) | CSF 14-3-3 immunoassay, MRI, EEG, PRNP gene testing, neuropathologically confirmed, diagnostic criteria for vCJD developed. |
| Zeidler, M et al, 1997, UK (7) | Cohort study | This study aims to report the psychiatric features of the first 14 Variant CJD cases in the UK. | First 14 cases of vCJD, (mean 29 years), prolonged duration of illness (median 14 months) | Necropsy is done in approximately 70% of all suspect cases |
| Zeidler M, Stuart G, UK 1997 (37) | Cohort study | This study aims to report the neurological features and diagnostic tests of the first 14 Variant CJD cases in the UK. | First 14 cases of vCJD, the mean age at onset of nvCJD was 29 years (range 16-48 years). The median duration of illness was long compared with sporadic CJD (14 and 45 months respectively). | Brain imaging, electroencephalography (EEG), cerebrospinal fluid (CSF) 14-3-3 protein, a test reported as both sensitive and specific for CJD. The diagnosis was established neuropathologically in all cases, four by cerebral biopsy and ten by necropsy. |
| Rajalingam P, 2023, Australia (49) | Retrospective case series | To report five cases of sporadic Creutzfeldt-Jakob disease presenting in Northern Tasmania. | 5 patients diagnosed with sporadic CJD | Clinical symptoms, EEG, CSF biomarkers (14-3-3, Tau), MRI, autopsy findings |
| Harrison KL, et al, 2022, USA (50) | Mixed methods cohort study | To identify targets for neuropalliative care interventions in sporadic Creutzfeldt-Jakob disease by examining characteristics of patients and sources of distress and support among former caregivers. | Median age of 70 (range: 60–86) years and disease duration of 14.5 months (range 4–41 months). Caregivers of deceased cohort participants were interviewed a median of 22 (range 11–39) months after patient death and had a median age of 59 (range 45–73) years. | Brain tissue pathology, cerebrospinal fluid (CSF) biomarkers of neuronal cell injury (i.e.14-3-3 Western blot, total-tau and neuron specific protein levels [45]), CSF RT-QuIC analysis [41], our internal review of brain MRI(s), and prion protein gene (PRNP) analysis. |

**Supplementary table 3: Symptoms list and tally**

| **AI- assisted Grouping** | **Symptom** | **Frequency of symptom reporting** | **Total per category** |
| --- | --- | --- | --- |
| Cognitive Disorders | Dementia | 4 | **26** |
|  | Cognitive decline | 5 |  |
|  | Memory loss | 3 |  |
|  | Confusion/disorientation | 2 |  |
|  | Amnesia | 1 |  |
|  | Forgetfulness | 3 |  |
|  | Frontal lobe syndrome | 2 |  |
|  | Loss of intellectual ability | 1 |  |
|  | Higher cortical function | 1 |  |
|  | Spatial disorientation | 1 |  |
|  | Cognitive collapse | 1 |  |
|  | poor concentration | 1 |  |
|  | Hemiospatial neglect | 1 |  |
| Behavioral, Psychiatric, and Emotional Symptoms | Psychiatric symptoms | 5 | **88** |
|  | Vegetative symptoms | 1 |  |
|  | Delusions/Paranoia | 5 |  |
|  | Personality change | 4 |  |
|  | Hallucination (auditory/visual) | 16 |  |
|  | Depression | 14 |  |
|  | Anxiety | 10 |  |
|  | Impaired emotion | 1 |  |
|  | Fear | 3 |  |
|  | Affective lability | 3 |  |
|  | Restlessness | 5 |  |
|  | Anergia | 1 |  |
|  | Dysphoria | 1 |  |
|  | Loss of interest | 1 |  |
|  | Withdrawal | 5 |  |
|  | Suicidal ideation | 2 |  |
|  | Persecutory delusions | 1 |  |
|  | Anhedonia | 1 |  |
|  | Nervousness | 1 |  |
|  | Tearfulness | 1 |  |
|  | Apathy | 1 |  |
|  | Lack of Drive | 2 |  |
|  | Agitation/aggression | 4 |  |
| Motor Impairments | Bradykinesia | 2 | **66** |
|  | Chorea | 5 |  |
|  | Choreoathetosis | 2 |  |
|  | Dystonia | 5 |  |
|  | Extrapyramidal signs | 4 |  |
|  | Extrapyramidal tremor | 2 |  |
|  | Myoclonus | 25 |  |
|  | Rigidity | 6 |  |
|  | Tremor | 9 |  |
|  | Cogwheel rigidity | 1 |  |
|  | Motor slowing | 1 |  |
|  | Athetosis | 2 |  |
|  | Upgaze paresis | 1 |  |
|  | Hypokinesia | 1 |  |
| Coordination, Balance, and Vestibular Issues | Ataxia | 21 | **66** |
|  | Dysdiadochokinesia/Bradydiadochokinesia | 1 |  |
|  | Falls | 1 |  |
|  | Gait disturbance | 8 |  |
|  | Unsteady gait/Ataxia | 2 |  |
|  | Dizziness | 11 |  |
|  | Vertigo | 7 |  |
|  | Bumping into objects | 1 |  |
|  | Dysmetria | 1 |  |
|  | Involuntary movements/jerks | 1 |  |
|  | Unsteadiness | 3 |  |
|  | Movement disorder | 9 |  |
| Visual and Sensory Disturbances | Blurred vision | 3 | **29** |
|  | Hemian optic loss of vision | 1 |  |
|  | Hemianopsia | 2 |  |
|  | Hemiospatial neglect | 1 |  |
|  | Metamorphopsia | 1 |  |
|  | Spatial agnosia | 1 |  |
|  | Visual disorder | 3 |  |
|  | Bizarre colour perception | 1 |  |
|  | Diplopia | 5 |  |
|  | Visual hallucination | 1 |  |
|  | Sensory deficit | 1 |  |
|  | Photophobia | 2 |  |
|  | Numbness | 1 |  |
|  | Paraesthesia | 1 |  |
|  | Sensory symptoms | 1 |  |
|  | Limb pain | 2 |  |
|  | Hyperacusis | 2 |  |
| Speech and Language Disorders | Aphasia | 9 | **38** |
|  | Apraxia | 4 |  |
|  | Alexia | 3 |  |
|  | Dysarthria | 12 |  |
|  | Global aphasia | 1 |  |
|  | Inability to communicate | 1 |  |
|  | Language disorder | 3 |  |
|  | Speech impairment | 1 |  |
|  | Agraphia | 4 |  |
| Autonomic and Sphincter Dysfunction | Autonomic disorders | 3 | **5** |
|  | Hyperhidrosis | 1 |  |
|  | Obstipation | 1 |  |
| Social symptoms | Social isolation | 3 | **4** |
|  | Loss of functional ability | 1 |  |
| Sleep Disorders | Insomnia | 4 | **25** |
|  | Sleep disturbance | 9 |  |
|  | Fatigue | 7 |  |
|  | Somnolence | 1 |  |
|  | Drowsiness | 2 |  |
|  | Hypersomulance | 1 |  |
|  | Exhaustion | 1 |  |
| Other Neurological | Seizure | 4 | **16** |
|  | Stroke-like presentation | 1 |  |
|  | Primitive reflexes | 2 |  |
|  | Headache | 8 |  |
|  | Impaired attention | 1 |  |
| Swallowing and nutrition | Dysphagia | 3 | **11** |
|  | Weight loss | 5 |  |
|  | Weight gain | 2 |  |
|  | Reduced appetite | 1 |  |
| **Total categories** | **Total symptoms** | **Total reported overall** | |
| 11 | 109 | 374 | |
